# Cartilage oligomeric matrix protein as a non-invasive biomarker for diagnosis of hepatocellular carcinoma in patients with liver cirrhosis 

**Published:** 2022

**Authors:** Hala A Abdel-Azeez, Hoda A Elhady, Abeer A Fikry

**Affiliations:** 1 *Clinical Pathology* *Department, Faculty of Medicine, Zagazig University, Zagazig, Egypt*; 2 * Internal Medicine Department, Faculty of Medicine, Zagazig University,* *Zagazig, Egypt*

**Keywords:** Cartilage oligomeric matrix protein, Hepatocellular carcinoma, Liver cirrhosis

## Abstract

**Aim:**

The current study purposed to evaluate serum COMP (Cartilage oligomeric matrix protein) as a diagnostic marker for HCC in patients with cirrhosis and to correlate it with other parameters of disease progression.

**Background:**

COMP is known to promote fibrosis in various tissues. Emerging evidence shows that COMP plays critical roles in tumor development. It can serve as a fibrosis and cancer biomarkers.

**Methods:**

The study included 24 subjects who serve as the healthy control, 24 cirrhotic patients without HCC, and 24 HCC patients with cirrhosis. All participants were subjected to liver function tests, AFP, calculation of fibrotic indices (APRI and FIB-4), and serum COMP by ELISA.

**Results:**

COMP was significantly increased in cirrhotic patients when compared to healthy controls and in HCC patients when compared to cirrhotic patients and healthy controls. A significant positive correlation was observed between COMP and APRI and FIB-4 in cirrhotic and HCC patients. Based on receiver operating characteristic (ROC) curve analysis, COMP had an **a**rea under curve (AUC) of 0.943 with 87.5% sensitivity and 79.2% specificity for diagnosis of HCC in cirrhotic patients. In combination with AFP, the sensitivity was increased to 100%.

**Conclusion:**

COMP might act as a promising non-invasive biomarker for HCC either alone or in combination with AFP. It was correlated with the degree of fibrosis and associated with advanced cancer staging.

## Introduction

 Hepatocellular carcinoma (HCC) is one of the most common malignancies and the third leading cause of cancer-related death worldwide (1). It represents the sixth most common cancer worldwide (2) and the fourth most common cancer in Egypt (3). The major risk factors for the development of HCC are chronic liver diseases such as hepatitis and liver cirrhosis. More than 80% of HCC arises from cirrhosis, which is an irreversible end result of chronic liver disease (4). HCC shows a growing incidence in Egypt, which may be the result of a shift in the relative importance of HBV and HCV as primary risk factors (5).

Diagnosis of early-stage HCC is difficult because of the lack of specific symptoms and the comparatively limited prognostic value of the serological and radiological approaches currently used for surveillance. The prognosis of HCC is generally poor due to the aggressive nature of the disease, synchronous liver decompensation, and sometimes limited availability of potential treatment choices (6). Screening of serum alpha-fetoprotein (AFP) levels and ultrasonography every 6 months appear to identify only a few cases with early-stage HCC, and therefore, its use is not prescribed by several international authorities (7).

Cartilage oligomeric matrix protein (COMP) is a pentameric particle found in various extracellular matrices (ECMs) that has been suggested to modulate collagen turnover by different systems, most notably by catalyzing collagen fibril formation (8). It is localized in the extracellular matrix of chondrocytes, synovial, tendons, and ligaments (9). COMP has been used primarily to assess the destruction of cartilage in patients with rheumatoid arthritis and osteoarthritis (10).

The process of viral hepatitis-cirrhosis-HCC is the main epidemiological development path of HCC in the world (11). Increased hepatic matrix stiffness resulting from the deposition and cross-linking of large amounts of matrix proteins not only occurs extensively in most solid tumors, but also promotes cell growth, motility, proliferation, metabolism, and tumor metastasis (12). Several publications have revealed that COMP is involved with the process of cirrhosis and HCC progression (13, 14). 

The current study aimed to evaluate serum COMP as a diagnostic marker for HCC in patients with cirrhosis and to correlate it with other parameters of disease progression. 

## Methods

**Study population**

This case control study was conducted on 72 subjects classified into three age- and sex-matched groups; 24 subjects served as the healthy control group; 24 cirrhotic patients without HCC and 24 HCC patients with cirrhosis served as the case groups. Patients were recruited from the in-patient sections and out-patient clinics of the Internal Medicine Department, Zagazig University Hospital from November 2019 to April 2020.

Liver cirrhosis is diagnosed by its characteristic findings on clinical examination, laboratory tests, and/or radiological imaging. HCC is diagnosed on the basis of the results of serological AFP determination, radiological imaging (i.e. liver ultrasound scan, triphasic computed tomography, or magnetic resonance imaging techniques) (15) or liver biopsy. Patients who had other malignancies or diseases that cause abnormalities in COMP, such as rheumatoid arthritis, osteoarthritis, other autoimmune rheumatic disease, systemic sclerosis, pulmonary fibrosis, cardiomyopathy, or skeletal dysplasia, were excluded from the study.

Written informed consent was obtained from all individual participants included in the study. Approval for the study was obtained from the Research Ethics Committee, Faculty of Medicine, Zagazig University.

**Methods**

All patients were subjected to history taking, clinical examination, and radiological imaging. Patients were classified according to Child-Pugh classification of severity of liver disease **(**16). Staging of HCC patients was carried out by Barcelona Clinic Liver Cancer (BCLC) (17).

Laboratory tests included liver function tests, AFP (Cobas 8000, Roche Diagnostics), platelets count (Sysmex XN-2000, Siemens), prothrombin time, INR (CA 1500, Sysmex), calculation of fibrotic indices (APRI (18) and FIB-4 (19)). Serum COMP levels were determined by enzyme-linked immunosorbent assay ELISA kit (Nova, China).

**Statistical Analysis**

Sample size was calculated by Epi-Info 6 at a confidence level 95% and power of test of 80%. Data analysis was performed using the SPSS (Statistical Package for the Social Sciences) version 20 (SPSS INC., Chicago, IL, USA) software. Categorical variables were described using their absolute frequencies, and chi-square test (χ 2) was used to compare frequencies of the data. The Kolmogorov-Smirnov (distribution type) and Levene (homogeneity of variances) tests were used to verify assumptions for use in parametric tests. Quantitative variables were described using their means and standard deviations or median and range. To compare two groups, the independent sample (t) test or the Mann-Whitney test (MW) was used, as appropriate. To compare more than two groups, the Kruskal Wallis test (KW) or analysis of variance ANOVA test (F test) was used, as appropriate. Correlations between two quantitative parameters were done by Pearson or Spearman Rank correlation, as appropriate. Multiple stepwise logistic regression analysis was used to identify independent predictor variables for COMP. Receiver operating characteristic (ROC) curve analysis was used to assess the best cutoff of studied parameters. Sensitivity, specificity, positive predictive value (PPV), negative predictive value (NPV), and accuracy were calculated. The level statistical significance was set at 5% (*p*<0.05). A highly significant difference was present if *p*≤0.001. 

## Results

Demographic and clinical data of the studied groups are presented in Table (1). Laboratory findings of the studied groups are presented in Table (2). COMP was significantly increased in the cirrhotic group when compared to the control group and in the HCC group when compared to the other groups (Table 2). There were significant positive correlations between COMP and APRI and FIB-4 in the cirrhotic and HCC groups, while COMP was not significantly correlated with other studied parameters (Table 3). COMP levels were not significantly different according to the Child-Pugh classification of severity of liver disease in cirrhotic and HCC patients (KW= -0.650 & MW= 0.391, respectively, and *p˃0*.05), while it was significantly increased in the BCLC stage (D) when compared with other stages (F=11.143, *p*<0.001). Regression analysis demonstrated that FIB-4, APRI, and BCLC are independently associated with COMP in HCC patients (Table 4).

**Figure 1:**
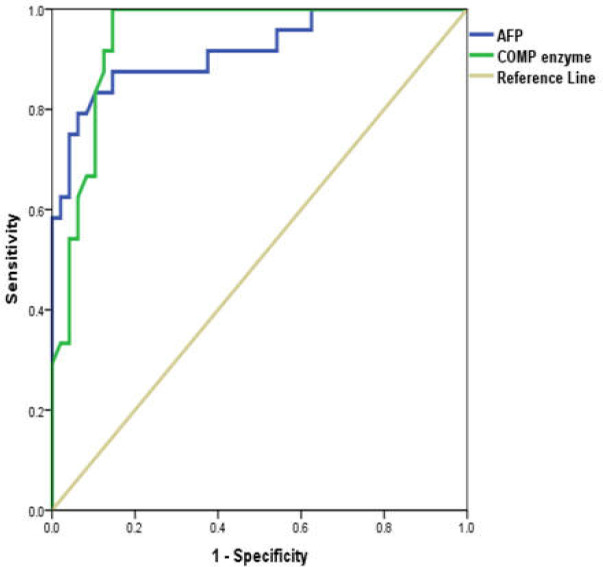
ROC curve showing performance of COMP and AFP in diagnosis of HCC

Area under ROC curve (AUC) of AFP was 0.971. A cutoff of 200 ng/mL for AFP in the diagnosis of HCC was chosen (20). At this cut-off, 17 out of 24 HCC patients were correctly diagnosed, while 2 out of 24 cirrhotic patients were falsely diagnosed as HCC (Table 5). ROC curve analysis of COMP as a diagnostic biomarker of HCC in cirrhotic patients revealed an AUC of 0.943. The best cutoff of COMP for the diagnosis of HCC is 15.6 ng/mL. At this cutoff, 21 out of 24 HCC patients were correctly diagnosed, while 5 of the cirrhotic patients were falsely diagnosed as HCC. By using both AFP and COMP in combination for the diagnosis of HCC at the same cutoff points, all HCC patients were correctly diagnosed (Figure 1 and Table 5).

**Table 1:** Demographic criteria and clinical data of the studied groups

P	HCC n=24	Cirrhosis n=24	Control n=24	Demographic & clinical data
0.675	15 (62.5) 9 (37.5)	13 (54.2) 11 (45.8)	12 (50) 12 (50)	Gender Male Female
0.140	60.83 ± 7.38	57 ± 5.82	58.83 ± 6.53	Age (years) *
0.221	2 (8.3) 22 (91.7) 0 (0)	0 (0) 23 (95.8) 1 (4.2)		Disease HBV HCV Combined
0.209	6.08 ± 2.99	4.92 ± 2.36		Disease duration(years) *
0.143	0 (0) 19 (79.2) 5 (20.8)	1 (4.2) 22 (91.7) 1 (4.2)		Child-Pough A B C
	1 (4.2) 2 (8.3) 2 (8.3) 11 (45.8) (33.3)			BCLC A1 A4 B C D

n: number of subjects; Data are represented as number (%)or mean± SD *;P˃0.05 is non significant

## Discussion

Although great progress has been made in HCC treatment, HCC prognosis remains poor (21). HCC is frequently diagnosed late in its course because of the absence of symptoms and the reluctance of many primary care physicians to provide surveillance for their high-risk patients (22); as a result, some patients had incurable disease at the time of diagnosis (23).

Alpha-fetoprotein has been the most widely used biomarker for hepatocellular carcinoma during the past several decades and is considered the gold standard by which other markers for the disease are judged (24). However, the clinical value of serum AFP to detect early HCC has been questioned because of its low sensitivity and specificity (25). These problems direct us to the necessity and urgency of identifying additional biomarkers with the potential of being used alone or complementary to AFP for HCC diagnosis.

**Table 2:** Laboratory findings of the studied groups

P	HCC n=24	Cirrhosis n=24	Control n=24	Laboratory finding
<0.001	1.44)0.6 – 16)	1.7)0.67 – 6.6)	0.45^∞ ^)0.3 – 0.8)	T.bilirubin (mg/dL)
0.001	0.65^∞^)0.1 – 8.5)	0.3 6^∞^)0.1 – 4.8)	0.2)0.0 – 0.2)	D.bilirubin (mg/dL)
<0.001	6.35 ± 0.7^∞^	6.88 ± 0.45	7.22 ± 0.62	T. protein (g/dL) *
<0.001	2.79 ± 0.5^∞^	3.15 ± 0.69^∞^	3.85 ± 0.49	Albumin (g/dL) *
<0.001	531)8 – 842)	57.5)15 – 850)	27^∞^)16 – 40)	ALT (U/L)
<0.001	50.5(25-527)	59(18 – 321)	25^∞^(19-35)	AST (U/L)
<0.001	244(130 – 480)	234(158-980)	95.5^∞^(68-114)	Alkaline phosphatase (U/L)
<0.001	121)29 – 426)	113)54 – 232)	239.5^∞^)152 – 393)	Platelets count/mL
<0.001	16.68 ±3.43^∞^	13.75 ± 1.47	13.04 ± 0.86	PT (seconds) *
0.001	1.45 ± 0.39^∞^	1.17 ± 0.21	1.13 ± 0.14	INR*
<0.001	337.5^∞^)7 – 3810)	52)6 – 261)	5.9)2.1 – 9.3)	AFP (ng/mL)
<0.001	2.18)1.16 –10.6 )	2.37(1.2 – 12.9)	0.26^∞^)0.15 – 0.45)	APRI
<0.001	3.54)0.58 – 18.8)	4.14)1.18 – 10.19)	1.28^∞^)0.71 – 1.85)	FIB-4
<0.001	19.24 ± 4.0^∞^	13.23 ± 3.6^∞^	7.7 ± 1.7	COMP (ng/ mL)*

n: number of subjects; Data are represented as median(range) or mean± SD *; ∞: significant with other group; p≤0.001 is highly significant

**Table 3:** Correlation between COMP levels and other parameters in patients groups

HCC group		Cirrhosis group		Laboratory finding
p	r	p	r	
0.673	-0.09	0.896	-0.028	Age
0.903	0.026	0.806	-0.053	Disease duration
0.054	0.398	0.289	0.226	T. bilirubin
0.118	0.328	0.531	-0.134	D. bilirubin
0.861	0.038	0.914	-0.023	T. protein
0.296	-0.233	0.139	-0.311	S. albumin
0.57	0.122	0.586	0.117	ALT
0.756	0.067	0.984	0.014	AST
0.396	0.181	0.173	0.288	Alkaline phosphatase
0.654	-0.096	0.208	0.267	AFP
0.454	-0.160	0.787	-0.081	Platelets count
0.359	0.196	0.851	-0.04	INR
<0.001**	0.633	0.004*	0.694	APRI
<0.001**	0.809	0.009*	0.654	FIB-4

*p<0.05 is significant; **p≤0.001 is highly significant

**Table 4:** Multiple stepwise regression analysis showing variables independently associated with serum COMP in HCC patients

	Unstandardized coefficient		Standardized coefficient	t	p
	Beta	Standard error	Beta		
APRI	1.112	0.320	0.694	3.477	0.004*
FIB-4	0.782	0.199	0.643	3.395	0.001**
BCLC	2.650	0.646	0.658	4.13	<0.001**

*p<0.05 is significant; **p≤0.001 is highly significant

**Table 5:** Performance of AFP and COMP in diagnosis of HCC in cirrhotic patients

Accuracy%	NPV%	PPV%	Specificity%	Sensitivity%	Parameter
81.3	75.9	89.5	91.7	70.8	AFP (ng/ml)
83.3	86.4	80.8	79.2	87.5	COMP (ng/ml)
83.3	100	75	66.7	100	AFP and COMP

Cartilage oligomeric matrix protein, a cartilage metabolism marker, is an extracellular matrix protein that modulates the cellular phenotype during tissue genesis and remodeling (26). COMP is known to promote fibrosis in skin, lung, and liver. Emerging evidence shows that COMP plays critical roles in the development of cancerous tumors, including those of breast cancer, colon cancer, and hepatocellular carcinoma (27).

In the present study, a highly significant increase in mean values of COMP was observed in cirrhotic patients compared to the controls. These findings are in agreement with those of Norman et al. (14) and Zachou et al. (28). Liver fibrosis is associated with major alterations in both quantity and composition of ECM (29), and COMP directly indicates ECM turnover in the liver (30). COMP increases type I collagen synthesis in the liver and stiffens the ECM contributing to cellular and organ dysfunction (31).

The current study demonstrated a positive correlation between COMP and fibrosis indices (APRI and FIB-4) in both the HCC and the cirrhosis groups. Regression analysis revealed that fibrosis indices (FIB-4 and APRI) are independently associated with COMP in HCC patients. Zachou et al. (30) reported that COMP levels are correlated positively with FIB-4 score in cirrhosis, and COMP was as good in detecting cirrhosis as the APRI and FIB-4 indices. Andréasson et al. (32) found that COMP is associated with the stage of liver fibrosis in chronic viral hepatitis C.

The current study revealed a highly significant increase in COMP levels in patients with HCC compared to cirrhotic patients and controls. Elevated COMP levels were associated with a higher risk of HCC development in cirrhotic patients (28). Li et al. (27) found that the level of COMP was increased in the serum of HCC patients compared to the healthy control, and the transition from premalignant lesions to HCC was associated with an increase in COMP serum levels. 

Xiao et al. (33) found that COMP was absent or rarely expressed in normal liver and liver cirrhosis tissues, but it was significantly overexpressed in HCC tissue samples when detected by northern blot and western blot analyses. When COMP mRNA and protein expression were localized within the cytoplasm of the tumor cells by in situ hybridization and immunohistochemistry analysis, COMP was highly expressed within the tumor cells of HCC, suggesting that COMP might play a role in the pathophysiology of HCC. Xiao et al. also showed that COMP was only weakly expressed in cirrhotic liver tissues, indicating that this gene might have a function early in the course of liver carcinogenesis.

ECM is a major component of tumor microenvironment and plays critical roles in cancer development and progression (34). In HCC, the metastatic microenvironment consists of activated hepatic stellate cells, extracellular matrix, and their secreted or released various cytokines to regulate tumor metastasis (35). COMP plays a very important role in the development and progression of HCC by activating the ERK and AKT signaling pathways in a CD36-dependent manner (27).

Patients with end-stage disease (BCLC stage D) have poor liver function (Child–Pugh class C). They are not candidates for transplantation and have marked cancer-related symptoms. They have a poor prognosis and require supportive care (36). We found that COMP levels are significantly increased in the BCLC stage (D) when compared with other stages. Regression analysis found that BCLC staging is independently associated with COMP in HCC patients. Li et al. (27) found that patients with a high level of serum COMP showed more unfavorable disease progression, such as higher incidence of vascular invasion, large tumor size, and tumor recurrence. Additionally, HCC patients with high serum COMP levels had poorer survival and disease-free survival rates. Studies have shown that COMP is present in human arteries and may play a role in the adhesion and migration of vascular smooth muscle cells during vasculogenesis (37, 38).

Norman et al. (14) assessed serum COMP in the outcome of HCC and cirrhotic patients in a long-term follow-up. They demonstrated that the presence of COMP in the sera of patients with chronic liver diseases is strongly associated with liver cirrhosis and that increased COMP levels appear to identify a subgroup of patients who are at increased risk of their disease progressing to HCC and liver-related mortality. Furthermore, 73.7% of patients who developed HCC during follow-up were COMP positive prior to the diagnosis of HCC. Although the presence of cirrhosis is clearly associated with an increased risk of disease progression, the detection of COMP in cirrhotic patients is a potentially useful marker to identify a subgroup of cirrhotic patients with a higher likelihood of developing HCC.

In the present study, serum AFP yielded an ROC-AUC of 0.917 for HCC versus liver cirrhosis. At a 200 ng/mL cutoff, sensitivity was 70.8% and specificity was 91.7%. AFP remains the most commonly used screening biomarker for HCC, although it suffers from poor sensitivity even at relatively low cutoffs and is even more limited in smaller tumors. Because AFP is secreted by regenerating hepatocytes as well as hepatic tumors, specificity is limited. AFP cannot be used reliably to distinguish malignant versus nonmalignant lesions, although specificity improves at higher levels. The presence of an elevated AFP, particularly an AFP that has increased from baseline, may alert the clinician to the possibility of HCC diagnosis (39). The current study revealed that COMP ROC-AUC for discriminating HCC patients from those with liver cirrhosis was 0.943, comparable with that of AFP. A cutoff of 15.6 ng/mL resulted in higher sensitivity than AFP (87.5%) and a specificity of 79.2%. When both AFP and COMP were used to diagnose HCC in cirrhotic patients, all HCC patients were correctly diagnosed (100% sensitivity). The lack of correlation between COMP and AFP in HCC patients encourages their use as complementary biomarkers in the diagnosis of HCC.

In conclusion, COMP might act as a promising non-invasive biomarker for HCC either alone or in combination with AFP. It is correlated with the degree of fibrosis and associated with advanced cancer staging. Further studies on a larger number of patients are recommended to confirm these results. In addition, serial measurements of COMP during the follow up of HCC patients are needed to evaluate its prognostic value.

## Conflict of interests

The authors declare that they have no conflict of interest.
